# Nanobubbles Adsorption and Its Role in Enhancing Fine Argentite Flotation

**DOI:** 10.3390/molecules30010079

**Published:** 2024-12-28

**Authors:** Shunde Yan, Xihui Fang, Guanfei Zhao, Tingsheng Qiu, Kaiwei Ding

**Affiliations:** 1 School of Resources and Environmental Engineering, Jiangxi University of Science and Technology, Ganzhou 341000, China; yanshunde2024@163.com (S.Y.); fangxihui202@163.com (X.F.); qiutingsheng@163.com (T.Q.); dingkaiwei2024@163.com (K.D.); 2 Jiangxi Provincial Key Laboratory of Low-Carbon Processing and Utilization of Strategic Metal Mineral Resources, Jiangxi University of Science and Technology, Ganzhou 341000, China

**Keywords:** nanobubbles, argentite, sodium diethyldithiocarbamate, adsorption, agglomeration

## Abstract

The efficient recovery of fine argentite from polymetallic lead–zinc (Pb–Zn) sulfide ore is challenging. This study investigated nanobubble (NB) adsorption on the argentite surface and its role in enhancing fine argentite flotation using various analytical techniques, including contact angle measurements, adsorption capacity analysis, infrared spectroscopy, zeta potential measurements, turbidity tests, microscopic imaging, scanning electron microscopy, and flotation experiments. Results indicated that the NBs exhibited long-term stability and were adsorbed onto the argentite surface, thereby enhancing surface hydrophobicity, reducing electrostatic repulsion between fine argentite particles, and promoting particle agglomeration. Furthermore, the NBs formed a thin film on the argentite surface, which decreased the adsorption of sodium diethyldithiocarbamate. Microflotation tests confirmed that the introduction of NBs considerably enhanced the recovery of argentite using flotation technology.

## 1. Introduction

Silver (Ag) is a precious metal predominantly found in association with other metals. Because of its strong affinity for sulfur, iron, and copper, Ag is commonly found in copper–lead–zinc-containing ores globally [1,2]. Although more than 200 Ag-containing minerals have been identified in nature, only approximately 10 varieties are viable for industrial production [3]. During the flotation process, the recovery of major metals such as copper, lead, and zinc is typically prioritized [4], which often leads to the insufficient enrichment of Ag-containing minerals and significant losses in tailings. Consequently, enhancing the efficient recovery of Ag-containing minerals has become a critical area of research.

Ag-containing minerals are typically characterized by fine dissemination size and low grades, making their recovery challenging. Various strategies, including grinding techniques and reagent optimization, have been used to address this issue. For example, Jiang et al. [5] improved Ag recovery from Pb concentrates by 5.59% through the regrinding of Pb middlings. Zhang et al. [6] enhanced the recovery of Ag by 2% using the flotation process, in which conventional ball mills were replaced with a ceramic-medium-stirred mill. Zhang et al. [7] developed a high-efficiency collector (A11) that increases the recovery of Ag from Pb concentrates by 8.61% during industrial production. The low flotation efficiency and recovery of fine Ag-containing mineral particles can be attributed to their small particle size, low mass, and high specific surface area [8,9].

Nanobubbles (NBs) are ultrafine bubbles with diameters < 1 µm (1000 nm) [10] and are characterized by unique properties such as [11,12] high stability [13,14,15] and large contact angles [16,17]. Recently, the application of NBs in mineral flotation has gained considerable attention. NBs enhance flotation by increasing the likelihood of collisions between the bubbles and mineral particles, thereby promoting the agglomeration of fine mineral particles and substantially enhancing recovery. Wang et al. [18] demonstrated that NBs reduce repulsion between particles, facilitate particle aggregation, and enhance the surface hydrophobicity in ultrafine molybdenite flotation. Zhang et al. [19] found that NBs compete with the benzohydroxamic acid (BHA) collector for adsorption on rutile surfaces, which reduces BHA adsorption but enhances mineral flotation through a synergistic interaction between the NBs and BHA, with NBs playing an auxiliary role during flotation. Despite some studies on understanding the role of NBs in enhancing the flotation of coal [20], metal ores [21], and oxidized ores [9], few studies exist on the combined effects of NBs and reagents on the flotation of associated Ag minerals. The underlying mechanism remains unclear.

Argentite is the primary Ag mineral in lead–zinc sulfide ores. This study examines the introduction of NBs into fine argentite flotation systems in the presence of sodium diethyldithiocarbamate (DDTC). The investigation focused on elucidating the unique characteristics of NBs, their adsorption behavior on the argentite surface, and their influence on particle aggregation and dispersion, as well as on argentite recovery. These findings provide valuable insights into the enrichment of fine Ag-containing minerals in lead–zinc sulfide ores.

## 2. Experimental

### 2.1. Materials and Reagents

**Figure 1:**
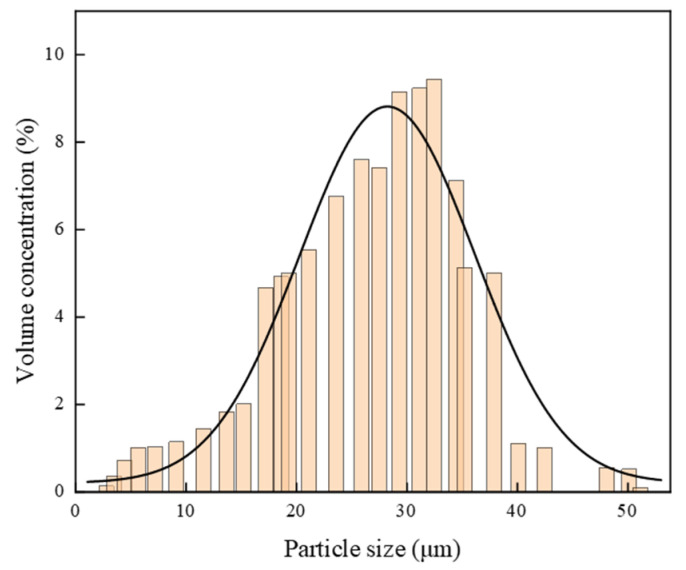
The distribution state of argentite particle sizes.

**Figure 2:**
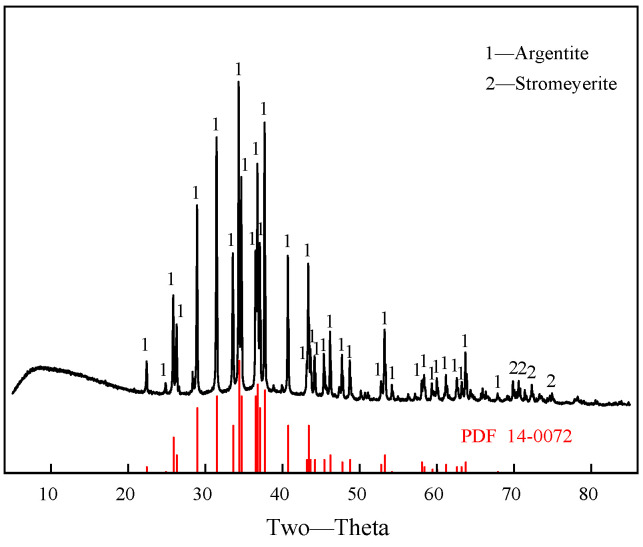
X-ray diffraction analysis results of argentite.

Pure argentite samples used in this study were sourced from the Tongbai silver mine, Tongbai County, Nanyang City, Henan Province, China. The samples were initially crushed and ground in an agate jar to obtain particles with sizes < 38 μm, and additional samples were prepared with particle sizes in the range of 38–74 μm. The particle size distribution, as shown in Figure 1, indicates that 90% of the argentite particles were smaller than 38 μm. To prevent the oxidation of the samples during extended exposure to air, the final samples were stored at −10 °C. X-ray diffraction (XRD) patterns are shown in Figure 2, in which the peak labeled “1” corresponds to that of argentite, consistent with the standard reference XRD card, confirming the high purity of argentite. A small peak corresponding to stromeyerite, another silver sulfide mineral, was also observed. Chemical analysis (Table 1) revealed an Ag content of 78.88% (compared to 87.10% for pure argentite) and an argentite purity of 90.56%, meeting the requirements for pure mineral flotation experiments.

**Figure 3:**
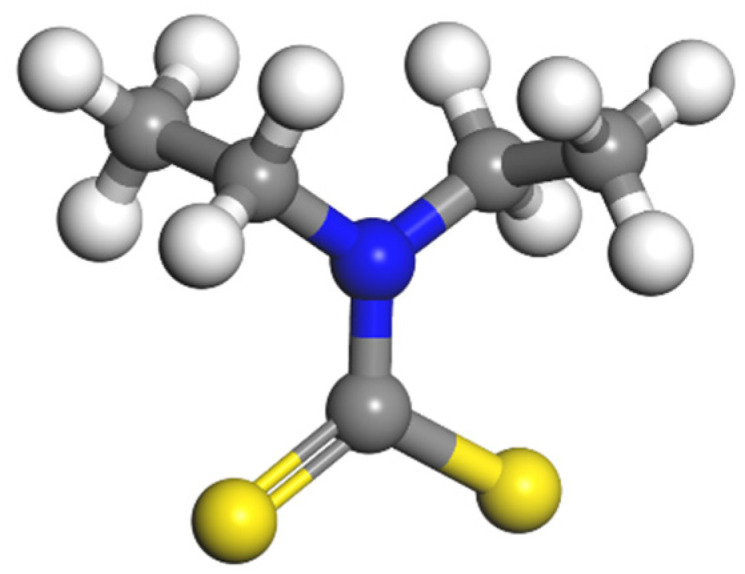
DDTC molecular structure.

All chemicals used in this study were of analytical grade. DDTC ((C_2_H_5_)_2_NCSSNa) was used as the collector, and its structure is shown in Figure 3. NaOH and H_2_SO_4_ were used as pH modifiers during the microflotation experiments. Deionized (DI) water with a resistivity of 18.25 MΩ⋅cm was used throughout the tests.

### 2.2. The Preparation of NBs

**Figure 4:**
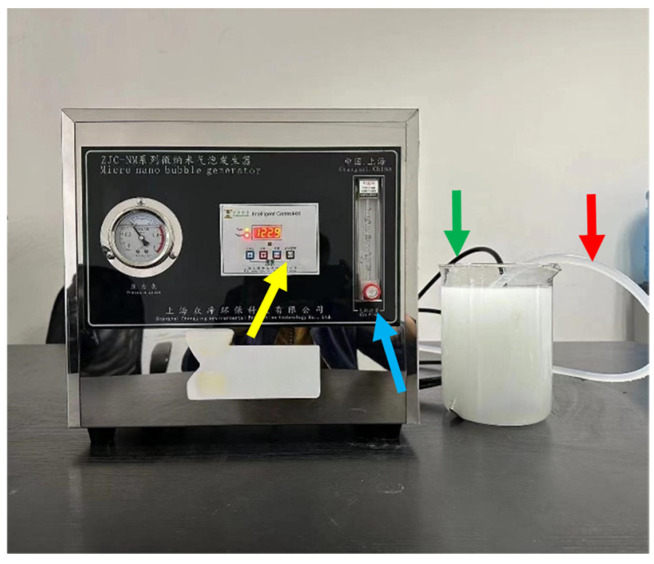
ZJC-NM-200L Micro-Nano Bubble Generator.

NBs were generated using a ZJC-NM-200L Micro-nano Bubble Generator (Shanghai Zong-jie Environmental Protection Technology corporation, Shanghai, China) (Figure 4). The device operates by intensely mixing water and air to create NBs via cavitation. Before operation, the water inlet tube was immersed in a beaker containing DI water. The tube, indicated by the red arrow, is the water inlet tube, whereas the tube indicated by the green arrow is the outlet tube for the NB solution. Upon activation, DI water and air were drawn into the generator through the inlet tubes, forming a high-speed gas–liquid mixture. The mixture was ejected through a specially designed nozzle under appropriate pressure, where hydrodynamic cavitation produced numerous NBs. The resulting solution initially appeared milky white. The DI water circulation time and inlet flow rate can also be adjusted. The intake volume was controlled using the knob indicated by the blue arrow, while the cavitation time was adjusted using the button indicated by the yellow arrow. After incubating for 2 min, larger bubbles ruptured and the milky white appearance disappeared, leaving a clear NB solution suitable for testing.

### 2.3. The Effect of Cavitation Time on NB Size

To evaluate the stability of the NBs and the effect of cavitation time on their size, we conducted experiments under a pressure of 0.35 MPa and an air inlet volume of 150 mL/min. A nanoparticle tracking analyzer (Malvern Panalytical Ltd., Worcester, UK; Nanosight NS300) was used to measure the size, concentration, and stability of the NBs at various cavitation times, enabling the determination of optimal conditions. Using nanoparticle tracking analysis, based on the principles of light scattering and Brownian motion, we measured particle sizes ranging from 30 to 1000 nm in solution [22]. This technique was used to assess the size distribution, average diameter, and density of the NBs. Each experiment was tested in triplicate, and the average values were calculated.

### 2.4. Zeta Potential Measurements

The adsorption of ions on the surface of the mineral particles alters their potential. Zeta potential measurements were used to assess changes in the potential on the surface of the mineral particles and their influence on the flotation process. The zeta potential of the argentite particles was measured under varying pH conditions using a NanoBrook 90Plus PALS potential analyzer. For each test, 50 mg of argentite particles (particle sizes < 5 μm) were dispersed in 40 mL of DI or NB water containing 1 mM KCl. The slurry was stirred using a magnetic stirrer, and the pH was adjusted as required. After 10 min of stirring, approximately 10 mL of the supernatant was extracted to measure the zeta potential. To investigate the effect of DDTC on the zeta potential of argentite, we adjusted the pH of the slurry following the same procedure. Briefly, 5 mg/L of DDTC was added, and the slurry was magnetically stirred for 10 min before being allowed to settle. Next, the supernatant was collected to measure the zeta potential. Each test was performed in triplicate, and the average value was calculated.

### 2.5. Turbidity Tests and Microscope Tests

Turbidity measurements were used to evaluate the dispersion and aggregation of the mineral particles. High turbidity indicated there was a large number of mineral particles in the suspension, indicating a dispersed pulp state. Low turbidity indicated that there were fewer mineral particles in the suspension, indicating an aggregated pulp state. The effects of the NBs on the aggregation and dispersion of argentite particles were assessed through turbidity and microscopic tests. For the turbidity tests, 1 g of argentite was mixed with 40 mL of DI and NB water in a beaker. The pH of the slurry was adjusted to the desired value before it was transferred into a 100 mL settling cylinder, in which it was left undisturbed for 3 min. Subsequently, 20 mL of the suspension was collected from a fixed position in the upper layer and analyzed using a WZS-188-type turbidimeter. Each measurement was repeated three times, and the average value was calculated.

For the microscopic examination, 0.5 g of argentite was dispersed in 100 mL of DI and NB water. No collector was added to the first two groups, whereas 5 mg/L DDTC was added to the last two groups. The slurry was stirred for 5 min, and the suspension was extracted from a fixed position and placed on a slide. The aggregation and dispersion of the argentite particles were observed under different conditions using a polarized light microscope (Leica M165C; Leica Camera AG, Wetzlar, Germany).

### 2.6. Contact Angle Tests

Regularly shaped argentite samples were prepared by cutting planes using a Unitom-type cutter. The cut samples were placed into abrasive molds, infused with an appropriate amount of epoxy resin, mixed with the curing agent, and allowed to cure for 24 h. Once set, the samples were polished to create fresh contact surfaces for testing.

The contact angles of the argentite samples were measured using a DSA30 contact angle meter (KRÜSS, A. Krüss Optronic GmbH, Hamburg, Germany) following the sessile drop method. Initially, the natural contact angle of the mineral surface was determined by depositing 2 μL each of DI and NB water on the sample surface using a microsyringe. The argentite samples were immersed in 5 mg/L DDTC solution for 30 min. The surfaces were rinsed with DI water to remove the residual DDTC and allowed to dry. Subsequently, DI and NB water were deposited on the DDTC-modified argentite surfaces to measure their contact angles.

### 2.7. Adsorption Capacity Measurements

**Figure 5:**
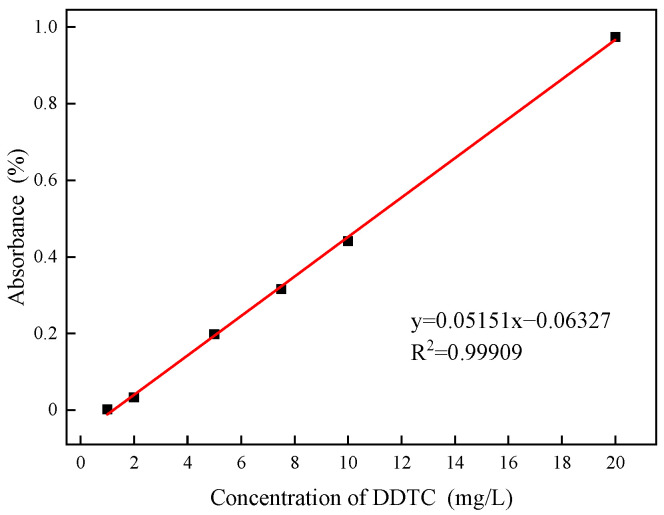
Adsorption standard curve of DDTC.

The adsorption of DDTC on the argentite surface was quantified using a T6 UV–visible spectrophotometer (Beijing PuXi General Instrument Co., Beijing PuXi General Instrument corporation, Beijing, China). Standard DDTC solutions with concentrations of 5, 10, 20, and 30 mg/L were prepared, and their absorbance values were measured to construct a standard curve (Figure 5). The linear relationship is described by the formula y = 0.05151x − 0.06327.

For each test, 2 g of argentite was placed in a 100 mL beaker and mixed with 40 mL of DI and NB water. The pH of the slurry was adjusted to 8, and DDTC was added at various concentrations. The slurry was stirred for 20 min to ensure an adequate reaction between the DDTC and the argentite particles. After stirring, the mixture was centrifuged, and the supernatant was transferred to the sample pool, where the absorbance was measured. Each test was repeated three times, and the average value was calculated. The DDTC concentration in the supernatant was determined using the linear formula. The adsorption capacity of the mineral surface for DDTC was calculated by subtracting the DDTC concentration in the supernatant from its initial concentration in the solution before the reaction.

### 2.8. Fourier Transform Infrared Spectral (FTIR) Analysis

FTIR spectroscopy is commonly used to investigate the interaction mechanisms between flotation agents and mineral surfaces. For the FTIR analysis, the sample was obtained from the same source as that used for the zeta potential measurements. A 2.0 g sample was added to 40 mL of DI or NB water, and the pH was adjusted. DDTC was then added at the same concentration, and the mixture was continuously stirred using a magnetic stirrer for 30 min to ensure full interaction between the DDTC and the mineral surface. The precipitate was washed three times with DI water, vacuum dried, and subjected to FTIR analysis.

### 2.9. Microflotation Tests

**Figure 6:**
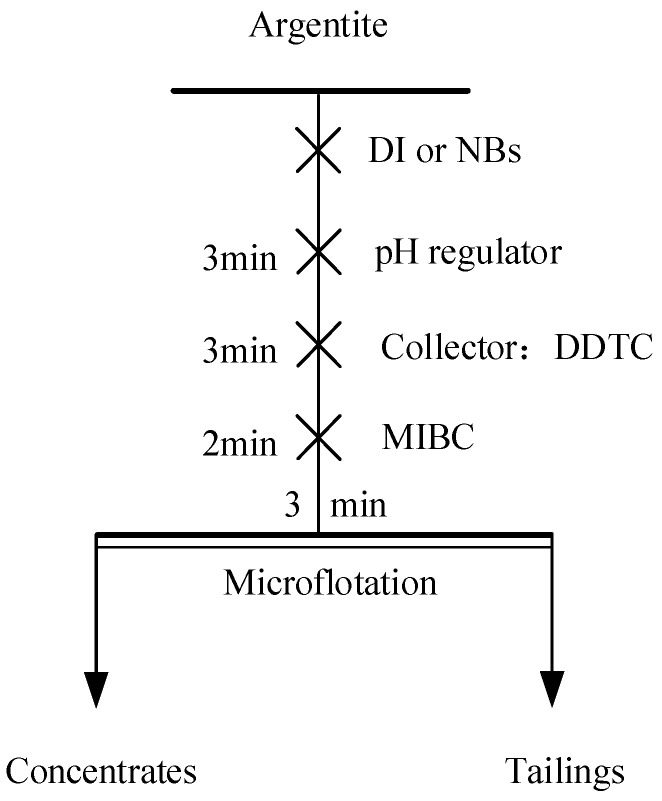
The flowsheet of the microflotation test.

Microflotation tests were conducted using an XFGCII-type flotation machine (Jilin Prospecting Machinery Factory, Changchun, China) with a stirring speed of 1500 rpm and a flotation tank volume of 40 mL (Figure 6). For each test, 2 g of argentite was placed in a clean flotation tank. DI and NB water were then added to adjust the liquid level. The slurry was stirred for 3 min to ensure the thorough dispersion of the mineral particles. pH regulators, collectors, and frothing agents were added according to the test protocol. The mixture was stirred for a specified duration. Flotation was initiated, and the froth was collected manually by scraping. After the completion of the flotation process, the froth and residual material in the flotation tank were filtered, dried, and weighed. The weights were used to calculate the recovery rate.

## 3. Results and Discussion

### 3.1. Characteristics of the NBs

**Figure 7:**
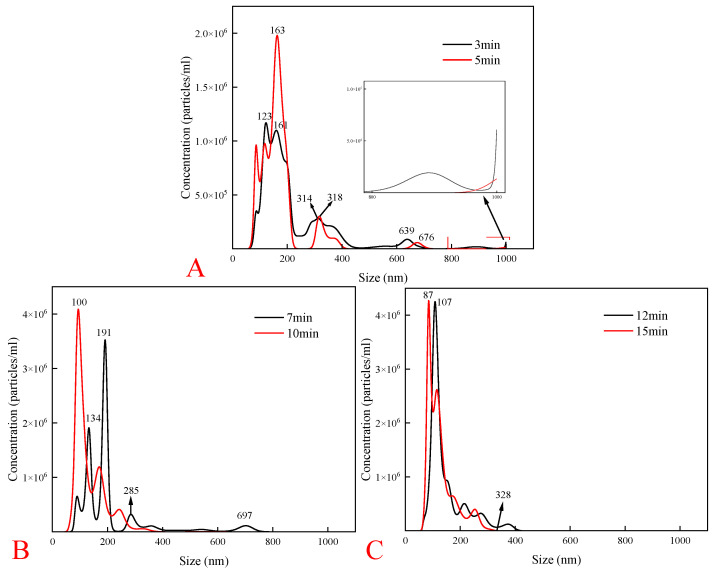
The effect of cavitation time on the concentration and size of the nanobubbles. Cavitation time: (**A**) 3mim, 5min; (**B**) 7mim, 10min; (**C**) 12mim, 15min.

To confirm the presence of NBs and evaluate the effect of cavitation time on their properties, we conducted experiments using a nanoparticle tracking analyzer. In this analysis, we measured the size distribution and concentration of the NBs at different cavitation times: 3, 5, 7, 10, 12, and 15 min (Figure 7).

As shown in Figure 7A, the initial stages of cavitation produced fewer NBs with a size of 1000 nm, indicating instability and turbulence in the solution and were consistent with the findings of Feng et al. [23]. As the cavitation time increased from 3 to 10 min (Figure 7B), the concentration of the NBs gradually increased, and the unstable bubbles broke apart and disappeared. The disappearance of 1000 nm-sized bubbles led to the formation of a large number of stable NBs that persisted in highly turbulent environments [24]. The statistical analysis revealed that the average NB sizes were 211 and 135 nm after 7 and 10 min of cavitation, respectively. Figure 7C shows that the size of the NBs continuously decreased with increasing cavitation time, with average sizes of 144 and 127 nm after 12 and 15 min of cavitation, respectively, indicating that prolonged cavitation improved the bubble distribution and stability. Similar findings were reported by Zhang et al. [25], who employed a comparable bubble generator and DLS technique to measure the average NB size, which was 192 nm after 10 min of cavitation. Based on these results, a cavitation time of 10 min was determined to be optimal.

### 3.2. The Effect of NBs on the Zeta Potential of Argentite

**Figure 8:**
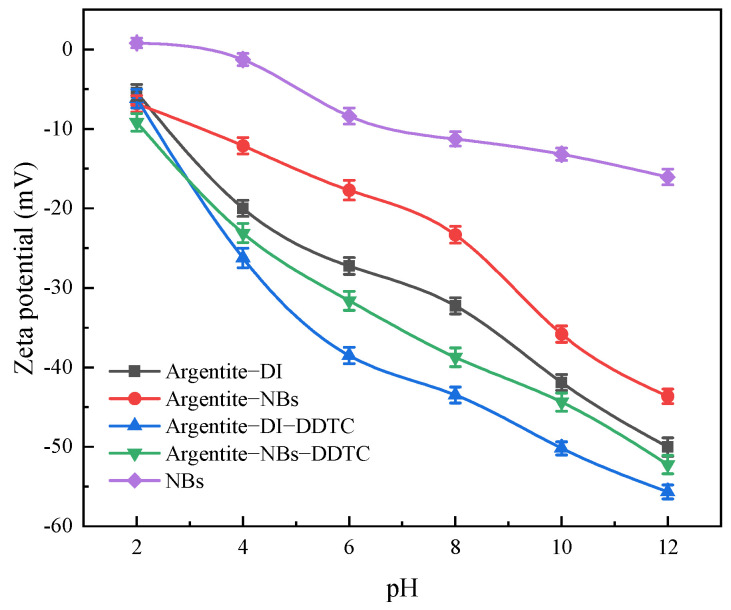
The zeta potential of argentite under different conditions.

Figure 8 shows the zeta potential of argentite particles under various pH conditions. In both DI and NB water, the zeta potential of the argentite surface exhibited a negative trend with increasing pH. The negative charge on the argentite surface can be attributed to the dissolution of the Ag ions by the alkaline pulp. However, in the presence of the NB water, the absolute zeta potential values of argentite were lower than those in the presence of DI water. This lowering of the zeta potential is likely due to the adsorption of the NBs on the argentite surface, which formed a protective thin film that prevented Ag ion dissolution. The decrease in the absolute zeta potential of argentite suggests weakened electrostatic repulsion between the argentite particles, facilitating their aggregation. Similar findings were reported by Zhou et al. [26], who observed that NB adsorption reduced the zeta potential of scheelite particles. Wang et al. [18] also demonstrated that NB adsorption decreased the zeta potential of molybdenite in the kerosene system.

In the pH range of 4–12, the zeta potential of the argentite surface exhibited a significant negative shift after the addition of DDTC. This shift can be attributed to the adsorption of negatively charged colloidal DDTC particles onto the argentite surface. However, the presence of NBs reduced the magnitude of the negative shift in the zeta potential after the addition of DDTC compared to flotation conducted with DI water. These observations indicated that the NBs were adsorbed on the argentite surface and formed a protective layer that hindered the adsorption of DDTC.

The absolute zeta potential of the argentite surface was lower in the presence of both NBs and DDTC than that in the conventional flotation system. This reduction in zeta potential enhanced the interparticle attraction and promoted the formation of stable hydrophobic flocs under the influence of the reagent. These flocs increased the likelihood of the attachment of fine argentite particles to bubbles while minimizing detachment during the flotation process.

### 3.3. The Effect of NBs on the Flocculation of Fine Argentite Particles

**Figure 9:**
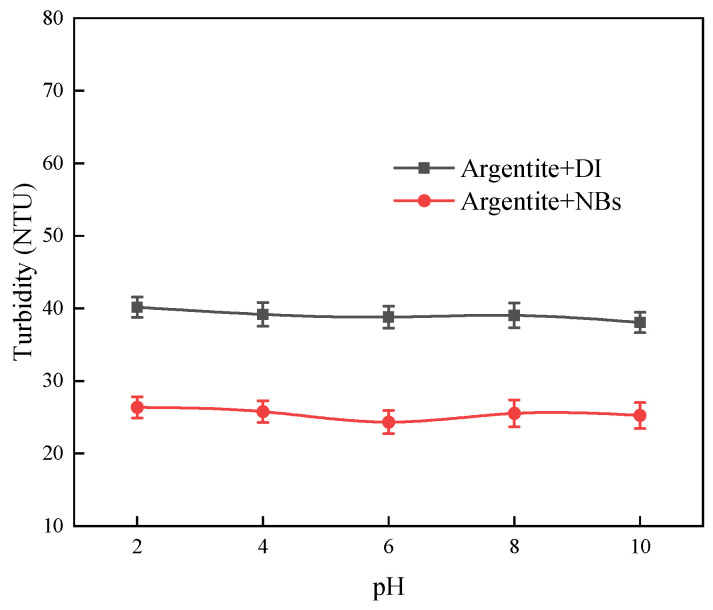
Turbidity of the argentite particles under different pH.

To investigate the effect of the NBs on argentite particles, we performed turbidity tests. Figure 9 shows the changes in the turbidity of the argentite slurry in DI or NB water at different pH levels. The turbidity of the argentite slurry was lower in NB water than in DI water, indicating that the argentite particles aggregated in the presence of NBs.

**Figure 10:**
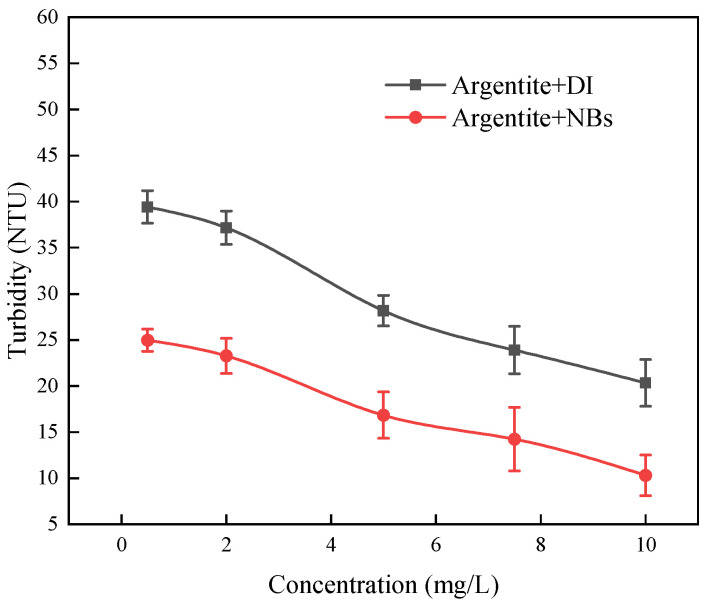
Turbidity of the argentite particles over different concentrations of DDTC.

Figure 10 shows the variation in the turbidity of the argentite slurry in DI or NB water at different DDTC concentrations. The turbidity of the argentite slurry decreased with increasing DDTC concentration, indicating that DDTC promoted the agglomeration of argentite particles. In addition, in the presence of NBs, the aggregation of the argentite particles was more pronounced. To further investigate the effect of NBs on the aggregation behavior of the argentite particles, we observed the aggregation and dispersion of the argentite particles under an optical microscope.

**Figure 11:**
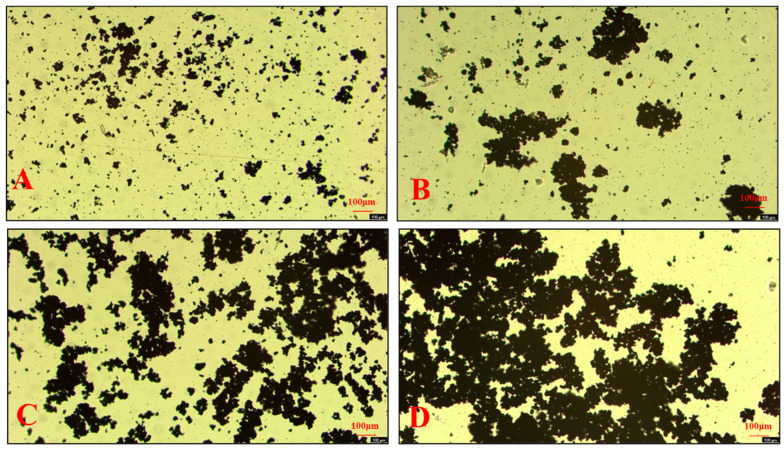
Optical microscopic observations of the fine argentite particles. (**A**) argentite + DI; (**B**) argentite + NBs; (**C**) argentite + DI + DDTC; (**D**) argentite + NBs + DDTC.

As shown in Figure 11, fewer aggregates of the argentite particles were formed in DI water (Figure 11A). However, the presence of the NBs facilitated the aggregation of argentite particles (Figure 11B). After adding 5 mg/L of DDTC to the pulp, the hydrophobic coalescence of DDTC led to the formation of irregular aggregates in DI water (Figure 11C), with a trace amount of dispersed fine particles. When both the NBs and DDTC were added, the aggregation of fine particles was significantly enhanced (Figure 11D). This effect can be attributed to the ability of the NBs to effectively overcome the electrostatic repulsion between argentite particles, resulting in the formation of large aggregates.

**Figure 12:**
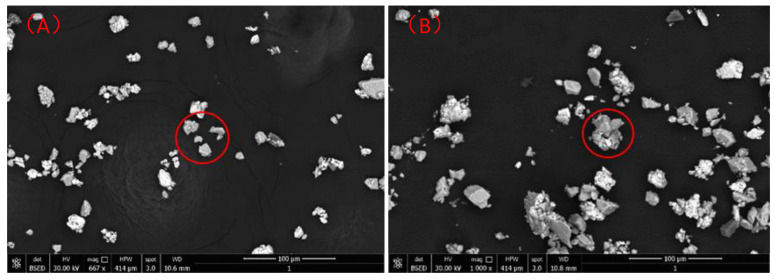
SEM images of the argentite particles (**A**) before and (**B**) after NB adsorption.

Figure 12 shows the scanning electron microscopy images of the argentite particles before and after NB adsorption. The red circle is shown in Figure 12A, the agglomeration of the argentite particles in DI water was minimal. However, the introduction of the NBs promoted the agglomeration of many dispersed argentite particles (Figure 12B red circle), resulting in the formation of large aggregates.

In summary, the introduction of NBs facilitated the aggregation of the argentite particles, primarily through the formation of bridges between them and inducing capillary forces [27]. Previous studies [28,29] have highlighted the significant role of bubble–particle interactions in reducing interparticle distances and increasing the likelihood of particle–bubble collisions during flotation processes.

### 3.4. The Effect of NBs on the Surface Wettability of Argentite

The contact angle is a key indicator of the mineral surface wettability, with a higher contact angle indicating greater hydrophobicity. Several studies have shown that NBs significantly increase contact angles. Ishida et al. [30] observed NBs on hydrophobic silicon wafers using an atomic force microscope and measured contact angles up to 160°. Additionally, NBs alter the hydrophobicity of mineral surfaces, enhancing macroscopic bubble contact angles, which increase the likelihood of mineral particle–air bubble collisions and facilitate particle flotation [31]. Zhou et al. [26] investigated the effects of collector dodecylamine (DDA) and NBs on the wettability of muscovite surfaces. The contact angle of the muscovite gradually increased with the increasing DDA concentration. Furthermore, the presence of NBs enhanced the hydrophobicity of the muscovite surface by promoting NB adsorption.

**Figure 13:**
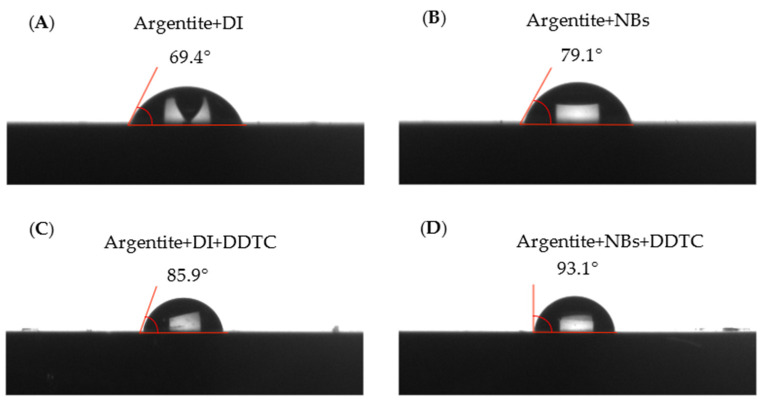
Contact angle of (**A**) argentite + DI; (**B**) argentite + NBs; (**C**) argentite + DI + DDTC; (**D**) argentite + NBs + DDTC.

Figure 13 shows the contact angle of the argentite surface under different conditions. The pulp used for contact angle measurement had a pH of 7. The contact angle measured using NB water was higher than that measured using conventional DI water. The introduction of the NBs increased the contact angle on the argentite surface by 9.7°. Furthermore, after the addition of DDTC, the increased adsorption of NBs enhanced the hydrophobicity of the argentite surface, resulting in a higher contact angle of 93.1° in NB water compared to 85.9° in DI water.

### 3.5. FTIR Analysis of the DDTC Adsorbed on the Argentite Surface

**Figure 14:**
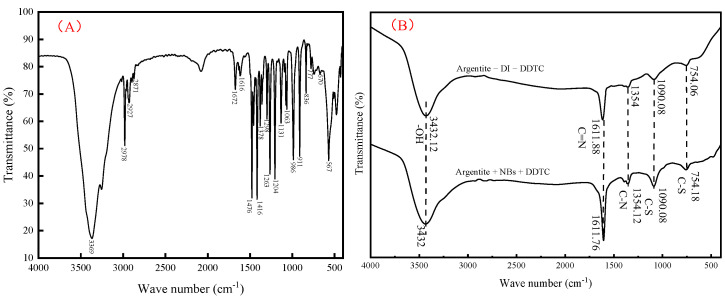
Infrared spectra of (**A**) DDTC and (**B**) argentite + DDTC.

The infrared spectra of the DDTC, argentite + DI water + DDTC, and argentite + NB water + DDTC systems were acquired, as shown in Figure 14.

Figure 14A shows the infrared spectra of DDTC. As shown in Figure 14B, compared with the infrared spectra of the argentite + DI + DDTC pulp system, the infrared spectra showed intense characteristic peaks at 754.06 and 1090.08 cm^−1^ (C–S stretching vibration peak), 1354 cm^−1^ (C–N stretching vibration), and at 1611.88 cm^−1^ (C=N stretching vibration). These results indicate that the adsorption of DDTC on the argentite surface was primarily chemical, and the adsorption stability of DDTC was higher in the NB flotation pulp system than in the DI flotation pulp system.

### 3.6. Adsorption Capacity Measurement

To evaluate the adsorption capacity of the argentite surface for different DDTC concentrations, we conducted measurements in both the DI and NB water systems.

**Figure 15:**
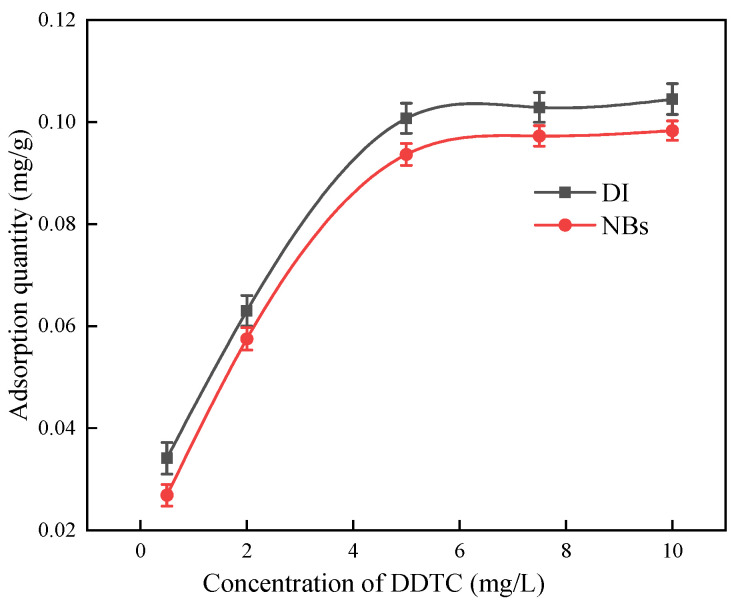
Effect of the concentration of DDTC on its adsorption on the surface of argentite.

As shown in Figure 15, the adsorption capacity of the argentite surface for DDTC initially increased with increasing DDTC concentrations in both systems. However, when the DDTC concentration exceeded 5 mg/L, the adsorption capacity plateaued, indicating that the mineral surface had reached saturation. A comparison between the conventional flotation and NB flotation systems revealed that the adsorption capacity of the argentite surface for DDTC was lower in the NB flotation system than in the conventional flotation system.

Wang et al. [32] examined the adsorption capacity of a calcite surface for sodium oleate in the presence of NBs and found that the NBs promoted the formation of calcite flocs. These flocs reduced the specific surface area of calcite particles, thereby decreasing their adsorption capacity for sodium oleate. Ren et al. [33] studied the effect of NBs on the flotation of fine cassiterite and observed that the amount of collector adsorbed on the cassiterite surface was always lower in the NB flotation system than in the conventional flotation system. However, the presence of NBs significantly enhanced the recovery of cassiterite.

In the presence of NBs, the adsorption capacity of the mineral surface for reagents was lower compared to that in conventional flotation. This reduction can be attributed to the occupation of adsorption sites by the NBs, which reduced the effective contact area between the minerals and collectors. Thus, the NB acts as a bridge between fine minerals and facilitates their aggregation [29]. This aggregation reduced the specific surface area of the particles, which affected the adsorption capacity of the argentite surface for DDTC.

### 3.7. Microflotation Test Results

This section explores the effect of NBs on the flotation behavior of fine argentite, building on previous findings. Single-mineral flotation tests were conducted to investigate the effect of the introduction of NBs on the recovery of fine argentite. DDTC was used as the collector, and MIBC functioned as the frothing agent. Initially, we conducted tests to establish the relationship between NB flotation and varying pH and identified the optimal pH for argentite flotation. Subsequently, under optimal pH conditions, we examined the effects of varying DDTC concentrations on the argentite flotation behavior. The outcomes of conventional and NB-enhanced flotation were compared.

The flotation tests were conducted with a fixed DDTC concentration of 5 mg/L and a MIBC concentration of 5 mg/L. NaOH and H_2_SO_4_ were used to adjust the pH of the slurry. Tests were performed in both the DI and NB water systems to investigate the effect of pH on argentite flotation.

**Figure 16:**
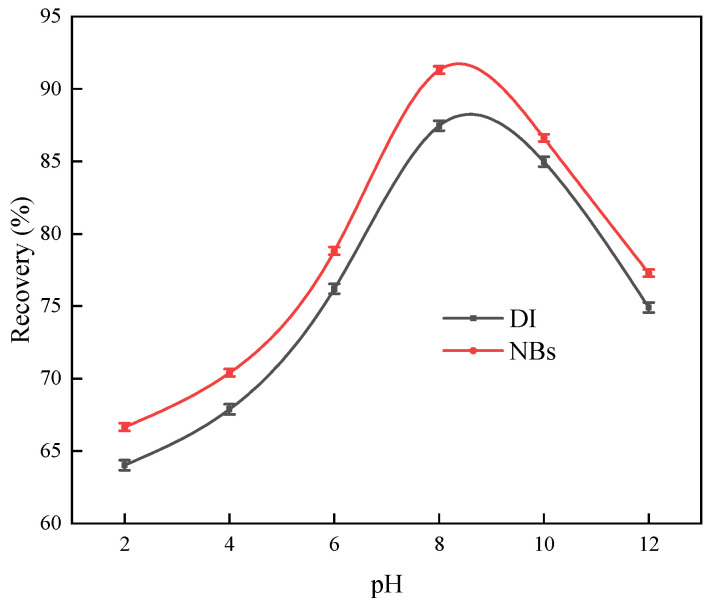
The recovery of argentite as a function of pH in the absence and presence of NBs (concentration of DDTC: 5 mg/L).

As shown in Figure 16, the argentite recovery initially increased with increasing pH and then decreased rapidly under alkaline pH conditions. The optimal flotation performance was observed under weakly alkaline conditions, with a recovery of 87.9% and 91.23% from the DI and NB water flotation systems, respectively, at pH 8. At pH > 10, the floatability of argentite decreased significantly. Throughout the pH range tested, the NB water flotation system consistently achieved higher recovery than the DI water flotation, enhancing the flotation of the fine argentite under comparable pH conditions. However, the presence of NBs had a minimal effect on the optimal pH for flotation.

**Figure 17:**
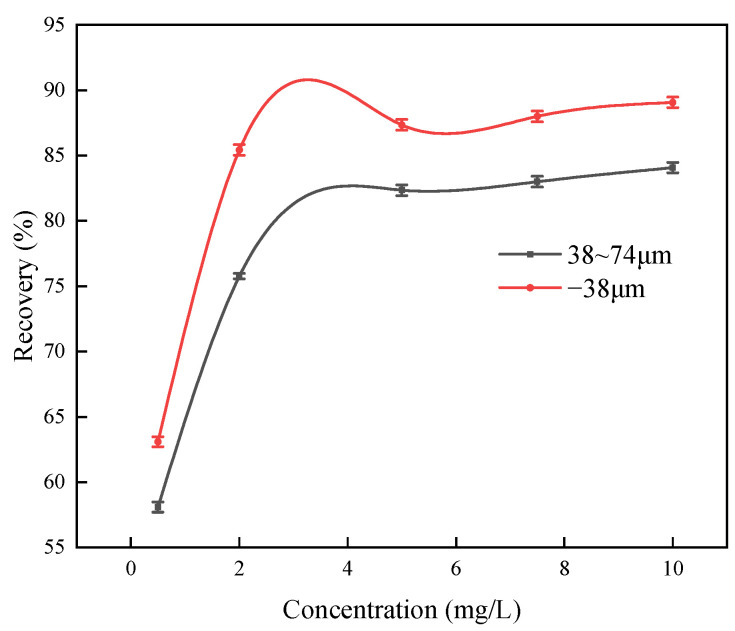
The flotation recovery of argentite with particle sizes 38 μm to 74 μm varies with DDTC concentration (pH = 8).

**Figure 18:**
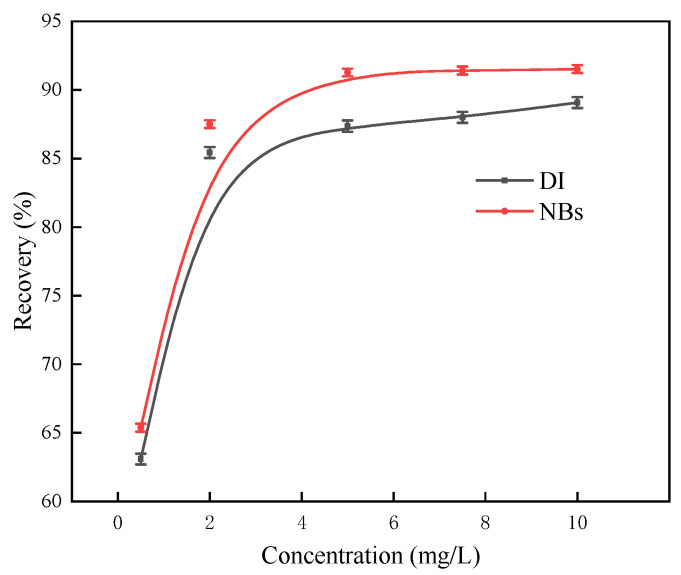
The flotation recovery of argentite as a function of DDTC concentration in the absence and presence of NBs (pH = 8).

Based on the tests conducted under varying pH conditions, a pH of 8 was identified as optimal for argentite flotation. Under these conditions, flotation tests were conducted on samples with particle sizes in the range of 38–74 μm to examine the relationship between the argentite floatability and DDTC concentration. The MIBC concentration was maintained at 5 mg/L (Figure 17). In addition, the effect of the DDTC concentration on the argentite flotation was evaluated in both the DI and NB water flotation systems while maintaining the MIBC concentration at 5 mg/L (Figure 18).

As shown in Figure 17, the recovery of argentite gradually increased with increasing DDTC concentrations, reaching a maximum of 84.07%. The primary factor contributing to the insufficient recovery of argentite was its large particle size, which reduced the efficiency of the separation processes by limiting interactions with the flotation reagents and hindering attachment to air bubbles. In practical applications, the fine grinding of silver minerals is essential for enhancing recovery.

As shown in Figure 18, a DDTC concentration of 0.5 mg/L resulted in a recovery of 63.65% from the DI flotation system and 65.29% from the NB flotation system. Higher DDTC concentrations significantly improved flotation recovery, which stabilized at 0.5 mg/L, and further increases had a minimal impact on recovery. Across the entire concentration range tested, NB flotation consistently outperformed conventional flotation, achieving approximately 3% higher recoveries.

In summary, NBs enhanced the recovery of fine argentite during flotation. For example, in the NB flotation system, the flotation recovery was 87.42% at a DDTC concentration of 2 mg/L, compared with 87.89% at a DDTC concentration of 5 mg/L in the DI flotation system. The introduction of NBs reduced the need for collectors, and NBs acted as a secondary collector for mineral particles during flotation, thereby reducing reagent costs [34]. Rahman et al. [35] reported similar findings when using NBs in the flotation of fine chalcopyrite, in which the introduction of NBs reduced the collector and frothing agent concentrations by 75% and 50%, respectively.

## 4. Conclusions

(1) Prolonged cavitation facilitated the formation of stable NBs with average bubble sizes ranging from 120 to 150 nm when the cavitation time exceeded 10 min;

(2) The NBs adsorbed on the argentite surface increased its hydrophobicity and induced significant aggregation of fine argentite particles, thereby enhancing the aggregation stability;

(3) The adsorption of more NBs on the argentite surface formed a thin film that reduced the amount of DDTC adsorbed on the argentite surface;

(4) Microflotation tests demonstrated that the presence of NBs enhanced the recovery of fine argentite. Furthermore, the use of NBs in fine argentite flotation technology can serve as a reference for improving the flotation processes of other fine minerals.

## Figures and Tables

**Table 1:** Analysis of the main chemical constituents of argentite (%).

Sample	Elemental Mass Concentration		Purity
	Ag	S	
Argentite	78.88	12.87	90.56

## Data Availability

Data are contained within the article.
